# Effect of Essential Oils on the Inhibition of Biofilm and Quorum Sensing in *Salmonella enteritidis* 13076 and *Salmonella typhimurium* 14028

**DOI:** 10.3390/antibiotics10101191

**Published:** 2021-10-01

**Authors:** Yuliany Guillín, Marlon Cáceres, Rodrigo Torres, Elena Stashenko, Claudia Ortiz

**Affiliations:** 1 Escuela de Biología, Universidad Industrial de Santander, Bucaramanga 680002, Colombia; yuliany2208193@correo.uis.edu.co; 2 Escuela de Medicina, Universidad Industrial de Santander, Bucaramanga 680002, Colombia; marlon2127899@correo.uis.edu.co; 3 Grupo de Investigación en Bioquímica y Microbiología, Bucaramanga 680002, Colombia; rodrigotorres4@hotmail.com; 4 Centro de Cromatografía y Espectrometría de Masas, CROM-MASS-CENIVAM, Facultad de Ciencias, Universidad Industrial de Santander, Bucaramanga 680002, Colombia; elenastashenko@gmail.com; 5 Escuela de Microbiología y Bioanálisis, Universidad Industrial de Santander, Bucaramanga 680002, Colombia

**Keywords:** essential oils, biofilm, *Salmonella*, antimicrobials, RT-qPCR

## Abstract

The emergence of multidrug-resistant microorganisms represents a global challenge that has led to a search for new antimicrobial compounds. Essential oils (EOs) from medicinal aromatic plants are a potential alternative for conventional antibiotics. In this study, the antimicrobial and anti-biofilm potential of 15 EOs was evaluated on planktonic and biofilm-associated cells of *Salmonella enterica* serovar Enteritidis ATCC 13076 (*S. enteritidis*) and *Salmonella enterica* serovar Typhimurium ATCC 14028 (*S. typhimurium*). In total, 4 out of 15 EOs showed antimicrobial activity and 6 EOs showed anti-biofilm activity against both strains. The EO from the *Lippia origanoides* chemotype thymol-carvacrol II (LTC II) presented the lowest minimum inhibitory concentration (MIC_50_ = 0.37 mg mL^−1^) and minimum bactericidal concentration (MBC = 0.75 mg mL^−1^) values. This EO also presented the highest percentage of biofilm inhibition (>65%) on both microorganisms, which could be confirmed by scanning electron microscopy (SEM) images. Transcriptional analysis showed significant changes in the expression of the genes related to quorum sensing and the formation of the biofilm. EOs could inhibit the expression of genes involved in the quorum sensing mechanism (*luxR, luxS*, *qseB*, *sdiA*) and biofilm formation (*csgA*, *csgB,* *csgD*, *flhD*, *fliZ*, and *motB*), indicating their potential use as anti-biofilm antimicrobial agents. However, further studies are needed to elucidate the action mechanisms of essential oils on the bacterial cells under study.

## 1. Introduction

Non-typhoidal salmonellosis is one of the most relevant zoonotic diseases across the globe due to the fact that it affects human and animal health [1]. Around 93 million people are infected by bacteria of the *Salmonella* genus and 155,000 die from this pathogen each year [2]. *Salmonella* is spread through the consumption of contaminated food or water and contact with infected people or animals [3]. *Salmonella* species are etiological agents of intestinal and systemic infections, the most frequent being gastroenteritis, bacteremia, an asymptomatic chronic carrier state, or localized infection [4]. Recently, the World Health Organization (WHO), the Food and Agriculture Organization of the United Nations (FAO), and the Center for Disease Control and Prevention (CDC) reported an increase in antibiotic resistance in *Salmonella* strains due to the indiscriminate use of conventional bactericides [5,6]. Consequently, some infections that were controlled a few decades ago now reoccur with multi-resistant microorganisms [7].

Antimicrobial multi-resistance is developed through various natural (family-specific) or acquired mechanisms (including horizontal gene transfer, gene deletions, and mutations) [8,9]. Several studies have shown that different *Salmonella* species are able to form biofilm through different types of chemical cell communication [10,11]. Thus, *Salmonella* is associated with different persistent hospital infections, especially in the immunocompromised population [12]. Biofilms are microbial communities made up of sessile cells irreversibly attached to a substrate, embedded in an extracellular polymer matrix produced by themselves [13]. Because the biofilm formed by *Salmonella* is resistant to environmental stress factors (e.g., low water activity or the presence of disinfectants), this state of cellular organization probably contributes to the survival of the microorganism outside the host and to the infection of new hosts [14]. Thus, the biofilm gives the microorganism increased resilience, inhibiting the bactericidal effect of antibiotics [15,16]. In addition, different studies have demonstrated the ability of *Salmonella* to form a biofilm on abiotic surfaces, such as plastic, rubber, cement, glass, and stainless steel [17,18]. Therefore, the search for new effective antimicrobial alternatives against resistant pathogens has received a significant increase in attention. During the last few years, numerous studies have been published, widely demonstrating the antimicrobial activity of EOs, which could have an important therapeutic potential due to their both high content and diversity of chemical compounds such as aldehydes, phenols, and terpenes [19,20,21]. This feature enables a wide spectrum of action on different microorganisms as well as multiple effects, such as the inhibition of quorum sensing (QS), the prevention of biofilm formation, and the inhibition of the growth of resistant microorganisms [22].

In this study, we aimed to determine the antimicrobial and anti-biofilm activity of 15 EOs on *Salmonella enterica* serovar Enteritidis ATCC 13076 and *Salmonella enterica* serovar Typhimurium ATCC 14028. In addition, the differential expression of some genes involved in quorum sensing and biofilm formation was measured by RT-qPCR to determine the potential anti-QS effect of EOs.

## 2. Results

### 2.1. Determination of the In Vitro Antimicrobial Activity of EOs on Planktonic Cells

The results of MIC_50_ and MBC are presented in Table 1. Four of the evaluated EOs (CM, LTC I, LTC II, and TV) presented inhibitory activity against the bacterial strains under study. The MIC_50_ values varied between 0.37 and 0.75 mg mL^−1^ and the MBC values between 0.75 and 1.5 mg mL^−1^. The EO showing the lowest MIC_50_ and MBC values against *S. typhimurium* and *S. enteritidis* was LTC II (MIC_50_ = 0.37 mg mL^−1^; CMB = 0.75 mg mL^−1^), which also had the highest content of thymol and carvacrol [23]. The LTC I EO showed a similar antimicrobial activity to LTC II on *S. enteritidis* (MIC_50_ = 0.37 mg mL^−1^; CMB = 0.75 mg mL^−1^), and both MIC_50_ and CMB values were increased in *S. typhimurium* (MIC_50_ 0.75 mg mL^−1^ and CMB 1.5 mg mL^−1^). The TV and CM EOs presented an MIC_50_ of 0.75 mg mL^−1^ and a CMB 1.5 mg mL−1 on both *Salmonella* strains. Regarding their chemical composition, EOs from LTC I, LTC II, and TV had a high content of phenolic compounds, such as thymol (the compound with the highest proportion in the three EOs) and carvacrol, as well as oxygenated monoterpenes and sesquiterpene hydrocarbons, such as *p*-cymene and trans-*β*-caryophyllene, respectively. Moreover, the EO from CM showed a high content of oxygenated monoterpenes, such as geraniol and linalool. The antimicrobial activities of the mentioned compounds have been mainly attributed to their interaction with the lipid bilayer of the cytoplasmic membranes, which is involved in the loss of integrity and the leakage of cellular material, such as DNA, ATP, or ions from bacterial cells [24].

### 2.2. In Vitro Inhibition of Biofilm Formation

**Figure 1:**
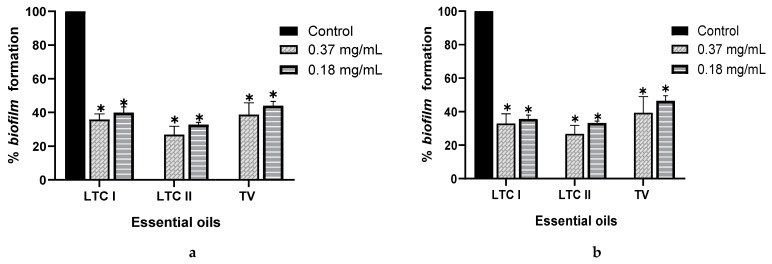
Effect of essential oils from *L*. *origanoides* (LTC I), *L. origanoides* (LTC II), and *Thymus vulgaris* (TV) on biofilm formation by *S. enteritidis* ATCC 13076 (**a**) and *S. typhimurium* ATCC 14028 (**b**). Data are represented as mean ± SD. ANOVA was used to show statistically significant differences with respect to the control. * *p* < 0.05.

Inhibition of biofilm was performed for the 15 EOs; however, essential oils that presented antimicrobial activity were evaluated at sub-inhibitory concentrations. The results are expressed as the percentage of inhibition (Table 2). EOs from LTC I, LTC II, and TV had the lowest minimum inhibitory concentrations in biofilm (MICB) on both *Salmonella* strains, with a percentage of inhibition of biofilm formation higher than 60% (Figure 1). For these EOs, the inhibition of biofilm formation could be attributed to the activity of the different oxygenated compounds and their ability to diffuse through the exopolysaccharide matrix (EPS), destabilizing it due to its intrinsic antimicrobial characteristics [25]. Other EOs, such as the AE of CM and TL, inhibited the formation of biofilm of *S. enterica* and *S. typhimurium* by up to 60%, although only the EO from CM exhibited antimicrobial activity. The LOF EO only showed anti-biofilm activity against *S. enteritidis*, inhibiting the formation of biofilm by 64.38% (MICB = 1.5 mg mL^−1^). Despite not showing any antimicrobial activity, the LOF and TL EOs could have the potential to be used as anti-biofilm agents.

### 2.3. Scanning Electron Microscopy

**Figure 2:**
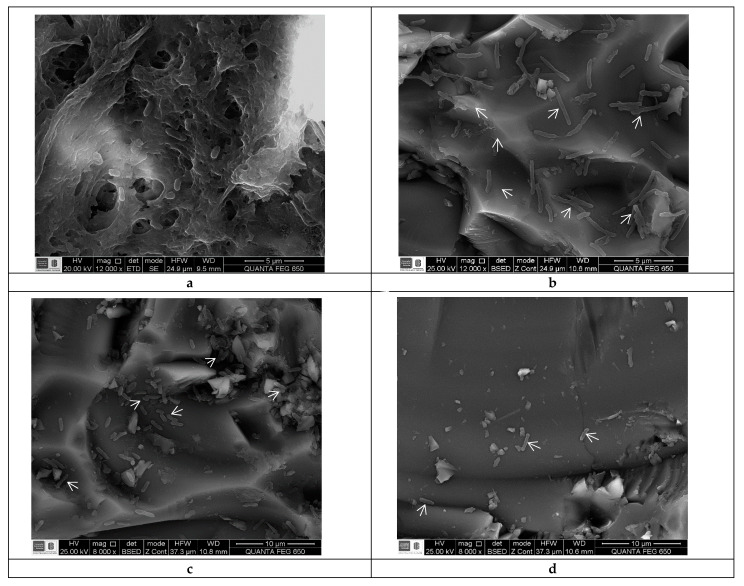
SEM micrographs of *S. enteritidis* and *S. typhimurium* biofilms formed on ground-glass coupons. (**a**) Non-treated *S. enteritidis* biofilm (control). (**b**) *S. enteritidis* biofilm treated with sub-inhibitory concentrations of EO from LTC II. (**c**) Non-treated *S. enteritidis* biofilm (control). (**d**) *S. typhimurium* biofilm treated with sub-inhibitory concentrations of EO from LTC II. Arrow indicates some bacteria.

*S. enteritidis* and *S. typhimurium* biofilms showed differences in cell density and morphology when treated with the LTC II EO compared to the untreated control. As shown in Figure 2, the untreated biofilm of *S. enteritidis* consisted of smooth-bacillary cells with an average size of 1102 ± 180 nm, embedded in a dense extracellular polysaccharide matrix (Figure 2a). The biofilm treated with the EO showed dispersed and morphologically irregular cells with an average size of 1450 ± 199 nm, as well as a decrease in the exopolysaccharide matrix (Figure 2b). A similar effect can be observed in the biofilm of *S. typhimurium*, in which the abundant formation of biofilm and regular bacillary morphologies of cells were observed with an average size of 1869 ± 395 nm (Figure 2c), whereas the biofilm treated with LTC II EO clearly shows cellular decrease and changes in bacterial morphology (Figure 2d).

### 2.4. QS and Biofilm Formation Gene Expression Analysis

**Figure 3:**
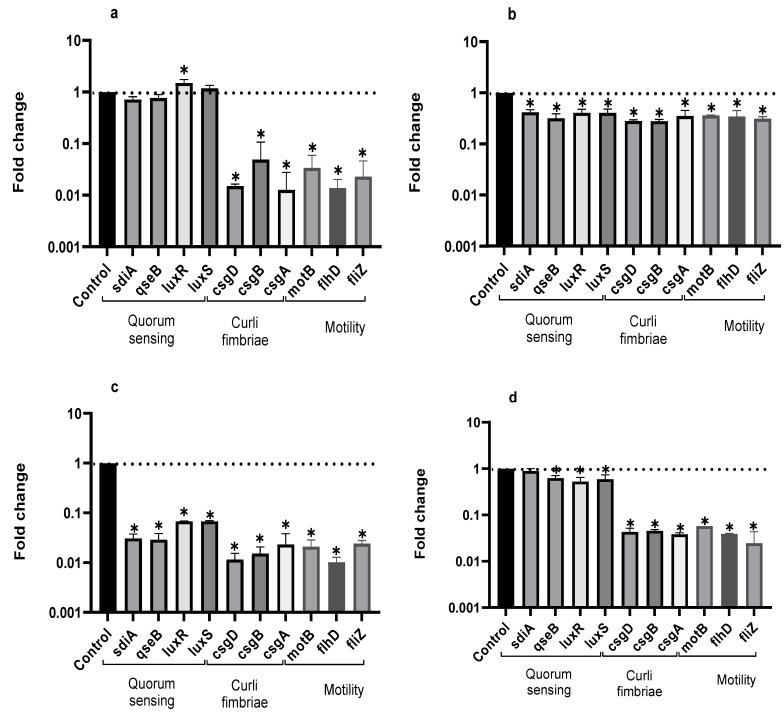
Expression profiles of QS genes in planktonic (**a**) and biofilm-associated cells (**b**) of *S. enteritidis*, and in planktonic (**c**) and biofilm-associated cells (**d**) of *S. typhimurium* treated or not treated with LTC II EO. The relative expression of the target genes was normalized to the *gst* reference gene. Bars represent standard deviation. ANOVA was used to show statistically significant differences with respect to the control. * *p* < 0.05.

RT-qPCR was used to evidence the influence of the EO from LTC II on the expression of genes involved in the QS signaling pathway and biofilm formation in sessile and planktonic cells of *Salmonella* (Figure 3). 

Compared to the control, EO from LTC II most markedly inhibited the expression of curli genes (*csgA*, *csgB,* and *csgD*) in planktonic and sessile cells for both microorganisms. In addition, LTC II EO notably down-regulated the expression of the genes involved in cell motility (swimming genes *motB*, *flhD*, and *fliZ*). Additionally, the genes involved in chemical cell communication were inhibited in *Salmonella* Typhimurium planktonic cells and in Enteritidis and Typhimurium biofilm cells, whereas the *luxR* gene in *Salmonella* Enteritidis planktonic cells was up-regulated. 

It has been previously reported that curli fimbriae are important for biofilm formation because they promote surface colonization and cell–cell interactions [26]. In addition, motility-related genes allow bacteria to develop active motility mechanisms that enable them to reach the surface of the culture medium and counteract hydrophobic interaction [27]. These results indicate that LTC II EO reduces the expression of genes related to primary adherence and motility, leading to the inhibition of the biofilm formation of *S. enteritidis* and *S. typhimurium*.

## 3. Discussion

Non-typhoidal salmonellosis infection is a worldwide problem. The lack of effective therapies, multi-resistance to antibiotics, and the ability to form biofilm are some of the main causes of the increase in the prevalence of bacterial diseases [28]. Therefore, it is necessary to find new antimicrobial agents that mitigate the resistance of high-incidence pathogens, such as *Salmonella*.

In this study, the antimicrobial and anti-biofilm effects of 15 EOs were evaluated on *Salmonella enterica* serovar Enteritidis ATCC 13076 and *Salmonella enterica* serovar Typhimurium 14028. It has been demonstrated that EO antimicrobial activity is caused by their chemical composition, the functional groups present in the active components, and synergistic interactions between the compounds [29]. EOs that obtained a higher antibacterial activity had a high content of phenolic compounds, such as thymol (EOs from LTC I, LTC II, and TV) and carvacrol (EOs from LTC I and LTC II) (Table 3). The mechanism of action of these compounds consists of the permeabilization of the bacterial cell membrane, followed by the loss of ions and membrane potential, which causes the collapse of proton pumps and the depletion of the adenosine triphosphate group of ATP, with consequent delays or inhibitions in microbial growth [30,31]. Other authors such as Sarrazin et al. have confirmed the antimicrobial effect of *L*. *origanoides* EO on food-borne microorganisms, attributing its antimicrobial activity to oxygenated compounds [32]. Additionally, they inferred that the antimicrobial activity of EOs may be the result of the interactions of all EOs components—thus, for example, *p*-cymene (biosynthetic precursor of these compounds) is not an efficient antimicrobial agent when it is individually used, but its presence in the EOs potentiates the action of other components of EOs [32].

Boskovic et al. determined the MIC of the TV EO on *S. enteritidis* ATCC 13076 and *S. typhimurium* ATCC 14028, obtaining similar results to those reported in this study (MIC = 0.32 mg mL^−1^) [33]. Moreover, CM EO also showed antimicrobial activity against *S. enteritidis* and *S. typhimurium*, which has been associated with a high content of oxygenated monoterpenes, such as geraniol [34], a compound that has been widely studied to determine its antimicrobial properties [35]. 

In our study, TV and CM EOs also inhibited the formation of the biofilm of *S. enteritidis* and *S. typhimurium* by 60% at sub-inhibitory concentrations. Further, in this study we report for the first time the anti-biofilm activity of the TL and LOF EOs against *Salmonella*. The antimicrobial and anti-biofilm activities of several of the major compounds of these EOs, such as estragole and 1–8 cineole, have been studied on various microorganisms [36].

One possible mechanism of action of EOs on biofilm has been attributed to the ability of EOs to diffuse through the EPS matrix, allowing interaction with bacterial membrane proteins and decreasing the binding of planktonic cells to surfaces [37,38]. Another reported mechanism is the reduction in motility and the interference of the production of adhesins or appendages such as curli proteins and flagella [38].

Transcriptional assays made it possible to establish that LTC II EO was able to inhibit the expression of genes related to the production of curli fimbriae, a key protein component of biofilms [39]. Both curli fimbrae and cellulose synthesis are co-regulated through the gene *csgD*. Thus, the inhibition of expression of *csgD* genes will inhibit cellulose production through the *adrA* regulator gene [26,40], which is consistent with the decrease in or absence of exo-polysaccharide matrix observed in SEM images (Figure 3b–d). Additionally, EOs inhibited the gene expression of *mot*B, *fliz*, and *flhD*, related to cell motility, reducing the bacterial capacity to produce biofilm. These results are consistent with those obtained by Inamuco et al., who proved that carvacrol was able to inhibit the motility of *Salmonella* Typhimurium at sub-inhibitory concentrations [41].

Moreover, LTC II inhibited the expression of the *sdiA*, *luxS*, and *luxR* genes implicated in QS. This effect could be related to the inhibition of the biosynthesis of signal molecules or the blocking of the reception of acyl homoserine lactone. At the same time, it is possible that molecules cause enzyme inactivation and the biodegradation of molecules involved in QS [42]. On the other hand, it is essential to carry out other omics studies to corroborate the metabolic pathways involved in QS.

These results prove the biological potential of EOs, mainly from LTC II, in the inhibition of sessile and planktonic cells from *S. enteritidis* and *S. typhimurium*, probably by means of the negative regulation of genes implicated in the production of proteins involved in both cell adherence and motility and QS. Moreover, EOs could also decrease the pathogenesis of *Salmonella* by the negative regulation of curli gene expression [43].

## 4. Materials and Methods

### 4.1. Plant Material

The EOs used in this study were previously reported by (Cáceres et al., 2020). Fifteen EOs were obtained from experimental crops at the CENIVAM Pilot Agro-industrial complex (N 07°08′442″; W 73°06′960″; 977 a.m.s.l.). The EOs were extracted by hydro-distillation in Clevenger-type equipment adapted to a Samsung microwave heating system, MS-1242zk (Seoul, Korea oven with an output power of 1600 W and a 2.4 GHz radiation frequency). The obtained EOs were dried over anhydrous sodium sulfate, weighed, and stored at 4 °C. All of the extractions were carried out in triplicate [44]. 

### 4.2. Bacterial Strains and Growth Conditions

*Salmonella enterica* serovar Typhimurium (*S. typhimurium*) ATCC 14028 and *Salmonella enterica* serovar Enteritidis (*S. enteritidis*) ATCC 13076 strains were obtained from the American Type Culture Collection (ATCC; Rockville, MD, USA). Before carrying out the antimicrobial and anti-biofilm experiments, both *Salmonella* strains were grown in M63 medium [45] at 37 °C. 

### 4.3. Determination of the In Vitro Antimicrobial Activity of EOs on Planktonic Cells

The antimicrobial effects of the 15 EOs were determined using the broth microdilution method (CLSI, 2015). The evaluation of the minimum inhibitory concentration (MIC) and minimum bactericidal concentration (MBC) was carried out as reported previously [23]. The inoculum used in the antimicrobial activity tests consisted of cultures prepared in M63 medium for 12 h at 37 °C with constant agitation at 200 rpm [45]; these were adjusted until a concentration of ~5 × 10^6^ CFU mL^−1^ was reached. Then, 100 µL of the inoculum was mixed with 100 µL of EO dissolved in 1% (*v*/*v*) DMSO in microplates for final concentrations of 0.18, 0.37, 0.75, and 1.5 mg mL^−1^. Microplates were incubated at 37 °C with constant agitation at 200 rpm. Microbial growth measurements were carried out at 595 nm every hour for 24 h in an ELISA microplate reader spectrophotometer (Biorad, imarck version 1.02.01, Hercules, CA, USA). After 24 h of culture in the presence of EO, 100 µL aliquots from each well were mixed with 900 µL of BHI medium in sterile tubes and incubated at 37 °C for 24 h (INE-500; Memmert, Schwabach, Germany). Then, a 10 µL aliquot was streaked on BHI agar plates to confirm the bactericidal effect. The MBC value was determined as the concentration at which 100% of the bacterial growth is completely inhibited compared to a control not treated with EOs. The antibiotic ofloxacin was used as a positive control of microbial inhibition for MIC and MBC. MIC is defined as the lowest concentration of EO at which an inhibition in the growth of the bacteria occurs.

### 4.4. In Vitro Inhibition of Biofilm Formation

The evaluation of the in vitro inhibition of biofilm formation was carried out according to the method described by Molhoek, with some modifications [46]. Bacterial strains were grown overnight in M63 medium at 37 °C and diluted in fresh medium (1:10). Then, 100 μL of cells was added to sterile 96-well flat-bottom polystyrene microplates containing sub-inhibitory concentrations (subMIC) of the EOs. Microplates were incubated at 37 °C for 24 h without shaking. Biofilm biomass was quantified using the crystal violet staining method. The microplates were washed three times with sterile phosphate buffer saline (PBS, 1 mM pH 7) to remove free-floating planktonic bacteria. Then, 200 μL of 0.4% (*w*/*v*) crystal violet was added to each of the wells for 15 min. Crystal violet excess was eliminated by three consecutive washes with sterile phosphate buffer saline (PBS, 1 mM pH 7) and 200 μL of 30% (*v*/*v*) acetic acid was then added to remove the adhered dye. The contents of each well were transferred to a new microplate to quantify the absorbance at 595 nm in an ELISA microplate reader [45]. 

### 4.5. Scanning Electron Microscopy 

Scanning electron microscopy (SEM) was used to visualize the effect of EOs on *S. typhimurium* ATCC 14028 and *S. enteritidis* ATCC 13076. An inoculum of each of the strains was prepared by culturing them in M63 medium for 12 h at 37 °C and orbital shaking at 200 rpm, until a concentration of ~5 × 10^6^ CFU mL^−1^ was reached. Then, 1 mL of bacterial inoculum was added to a 40 mL aerated batch bioreactor containing 9 mL of fresh M63 medium, a 2 × 5 cm ground-glass coupon, and the EO at a sub-inhibitory concentration. Cultures were conducted for 24 h at 37 °C. Coupons were washed three times with 0.1 mM of phosphate buffer after incubation to eliminate planktonic cells. Biofilm-associated cells were fixed with 2.5% (*v*/*v*) glutaraldehyde for 60 min and dehydrated using an isopropanol gradient (10 to 95%) for 10 min [47]. Finally, the coupons were observed in a Quanta 650 FEG electron microscope (FEI, Hillsboro, OR, USA) equipped with an Everhart Thornley ETD image detector.

### 4.6. RNA Extraction and Transcriptional Analysis 

The formation and inhibition of biofilm assays were carried out in aerated bioreactors containing 40 mL of M63 medium, a 2 × 5 cm ground-glass coupon, and the EO at a sub-inhibitory concentration, which were inoculated with ~5 × 10^6^ CFU mL^−1^ of each of the strains. Cultures were conducted for 24 h at 37 °C. The coupon was washed with sterile phosphate buffer saline (PBS, 1 mM pH 7) to remove planktonic cells and biofilm-associated cells were then harvested for RNA extraction using sterile spatulas. Total RNA was isolated using a PureLink RNA Mini Kit (Ambion, Life Technologies, Carlsbad, CA, USA). The RNA purity and quality were verified using an NP80 Nanophotometer (IMPLEN, München, Germany) and cDNA synthesis from RNA was performed with the RevertAid™ H Minus First Strand cDNA synthesis kit (Fermentas, Thermo Fisher Scientific, Madison, WI, USA).

RT-qPCR assays were carried out using the Sybr green-based Luna^®^ Universal qPCR Master Mix (New England Biolabs, Ipswich, MA, USA) in a CFX96™ Real-Time PCR Detection System (Bio-Rad, Hercules, CA, USA). According to the manufacturer’s instructions, reactions were prepared with a final volume of 20 μL containing 1X master mix, 0.25 μM of each primer, 100 ng template cDNA, and water. The amplification programs consisted of an initial denaturing at 95 °C for 1 min, followed by 40 cycles of 95 °C for 15 s, 55–60 °C for 45 s. Negative controls (NTC) consisted of omitting any cDNA template from the reaction. The relative expression of the genes involved in *Salmonella* QS cell signaling, such as *sdiA*, *luxR* (encoding the autoinducer 1—AI-1), *luxS* (encoding the autoinducer 2—AI-2), and *QseB* (encoding the response regulator); curli genes, such as *csgA* (curli subunit, major curli subunit), *csgB* (curli nucleator protein curli), and *csgD* (transcriptional activator for csgBA); motility genes *fliZ* (flagellar biosynthesis regulator *FliA*), *flhD* (flagellar biosynthesis regulator), and *motB* (flagellar rotation generator), were determined using the glutathione transferase (*gst*) and 6-phosphoglucanate dehydrogenase (*gnd*) genes as a reference for normalization. The Primer3 software [48] and OligoCalc [49] were used to design gene-specific primers, as shown in Table 4. A gene expression and efficiency calculation was performed using the 2^−ΔΔCt^ method [50].

### 4.7. Statistical Analysis

All results are expressed as means and their respective standard deviations for each of the assays. The Kolmogorov–Smirnov test [51] was used to verify the normality of the data and a maximum F test to test the homogeneity of variance (ANOVA). All statistical analyses were performed on the R platform [52], considering *p* < 0.05 as statistically significant. Significant changes are indicated by asterisks in the figures.

## 5. Conclusions

EOs with a higher content of oxygenated compounds exhibited greater antimicrobial and anti-biofilm activity than the other EOs used against *S. enteritidis* and *S. typhimurium*. EO isolated from the *L. origanoides* chemotype thymol-carvacrol (LTC II) showed the highest antimicrobial activity against both *Salmonella* strains, whereas LTC I and LTC II EOs displayed the highest anti-biofilm potential. These differences in antimicrobial activity can be attributed to changes in the expression of genes involved in biofilm formation, demonstrating that the EOs used in this study not only inhibited QS signaling, which would affect the processes of the initiation, maturation, and dispersion of the biofilm, but also modified the expression of genes involved in the adhesion, motility, and. production of EPS. Finally, the EOs used in this study represent a promising alternative for both microbial control and therapeutic treatment against pathogenic resistant bacteria. However, further studies are needed in order to elucidate the mechanisms of action of the essential oils on the bacterial cells under study.

## Figures and Tables

**Table 1:** Minimum inhibitory concentration (MIC_50_) and minimum bactericidal concentration (MBC) of the 15 EOs on *S. typhimurium* ATCC 14028 and *S. enteritidis* ATCC 13076. Each experiment was carried out in triplicate. Data are represented as mean ± SD.

Essential Oils		Antimicrobial			
		*S. typhimurium*		*S. enteritidis*	
Code	Plant	MIC_50_	MBC	MIC_50_	MBC
		mg mL^−1^			
LACA	*Lippia alba* (Mill.) (carvona)	>1.5	>1.5	>1.5	>1.5
LACI	*L. alba* (citral)	>1.5	>1.5	>1.5	>1.5
CN	*Cymbopogon nardus* (L.)	>1.5	>1.5	>1.5	>1.5
CM	*C. martini* (Roxb.)	0.75 ± 0.07	1.5 ± 0.01	0.75 ± 0.02	1.5 ± 0.04
CF	*C. flexuosus* (Nees ex Steud.)	>1.5	>1.5	>1.5	>1.5
LTC I	*L. origanoides* (Kunth) (thymol-carvacrol I)	0.75 ± 0.16	1.5 ± 0.05	0.37 ± 0.02	0.75 ± 0.03
LTC II	*L. origanoides* (thymol-carvacrol II)	0.37 ± 0.08	0.75 ± 0.06	0.37 ± 0.03	0.75 ± 0.02
LOF	*L. origanoides* (phellandrene)	>1.5	>1.5	>1.5	>1.5
RO	*Rosmarinus officinalis* (L.)	>1.5	>1.5	>1.5	>1.5
SO	*Salvia officinalis* (L.)	>1.5	>1.5	>1.5	>1.5
SG	*Swinglea glutinosa* (Blanco)	>1.5	>1.5	>1.5	>1.5
TL	*Tagetes lucida* (Cav.)	>1.5	>1.5	>1.5	>1.5
TV	*Thymus vulgaris* (L.)	0.75 ± 0.05	1.5 ± 0.03	0.75 ± 0.02	1.5 ± 0.05
SV	*Satureja viminea* (L.)	>1.5	>1.5	>1.5	>1.5
CO	*Cananga odorata* (Lam.)	>1.5	>1.5	>1.5	>1.5

**Table 2:** Effect of EOs on biofilm formation by *S. typhimurium* ATCC 14028 and *S. enteritidis* ATCC 13076. Inhibition percentages were calculated with respect to the control (biofilms not treated with EOs). Each experiment was carried out in triplicate. Data are represented as mean ± SD.

Essential Oils		Anti-Biofilm			
		*S. typhimurium*		*S. enteritidis*	
Code	Plant	MICB	Inhibition (%)	MICB	Inhibition (%)
		mg mL^−1^			
LACA	*Lippia alba* (Mill.) (carvona)	>1.5	ND	>1.5	
LACI	*L. alba* (citral)	>1.5	ND	>1.5	ND
CN	*Cymbopogon nardus* (L.)	>1.5	ND	>1.5	ND
CM	*C. martini* (Roxb.)	0.37 ± 0.02	50.87 ± 0.04	0.37 ± 0.10	46.72 ± 0.05
CF	*C. flexuosus* (Nees ex Steud.)	>1.5	ND	>1.5	ND
LTC I	*L. origanoides* (Kunth) (thymol-carvacrol I)	0.18 ± 0.01	62.18 ± 0.06	0.18 ± 0.01	61.32 ± 0.08
LTC II	*L. origanoides* (thymol-carvacrol II)	0.18 ± 0.18	66.64 ± 0.08	0.18 ± 0.08	65.64 ± 0.01
LOF	*L. origanoides* (phellandrene)	>1.5	ND	1.5 ± 0.06	64.38 ± 0.03
RO	*Rosmarinus officinalis* (L.)	>1.5	ND	>1.5	ND
SO	*Salvia officinalis* (L.)	>1.5	ND	>1.5	ND
SG	*Swinglea glutinosa* (Blanco)	>1.5	ND	>1.5	ND
TL	*Tagetes lucida* (Cav.)	1.5 ± 0.07	53.00 ± 0.10	1.5 ± 0.02	67.02 ± 0.23
TV	*Thymus vulgaris* (L.)	0.37 ± 0.7	59.33 ± 0.14	0.37 ± 0.10	68.52 ± 0.21
SV	*Satureja viminea* (L.)	>1.5	ND	>1.5	ND
CO	*Cananga odorata* (Lam.)	>1.5	ND	>1.5	ND

ND—not detected.

**Table 3:** Major chemical constituents present in the EOs assessed. Relative amount of each metabolite is reported as a percentage (%).

Code	Plant Species	Identified Metabolites (%)
**CM**	*C. martini* (Roxb.)	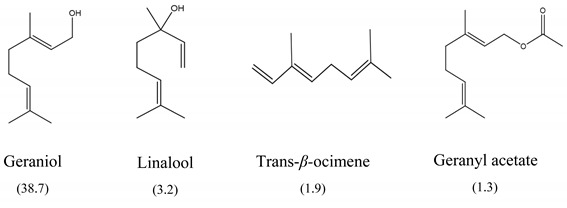
**LTC I**	*L. origanoides* (Kunth) (thymol-carvacrol I)	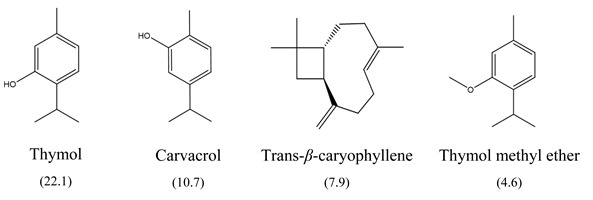
**LTC II**	*L. origanoides* (thymol-carvacrol II)	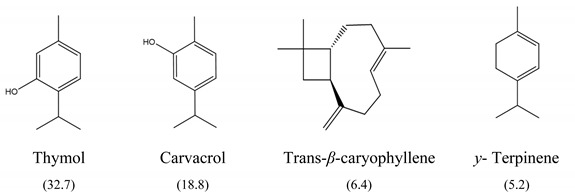
**TV**	*Thymus vulgaris* (L.)	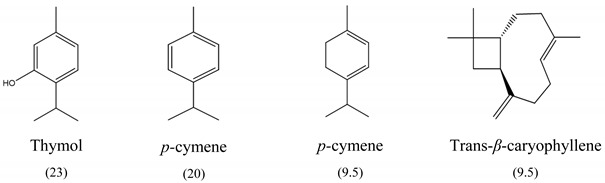

**Table 4:** List of primers used for amplification.

Genes	Forward (5′–3′)	Reverse (3′–5′)
*gnd*	ACGCAGAAAACGCTGGTATC	CCACTCGGTATGGAAAATGC
*gst*	TGTGGATGAGTCGCTTTCAG	GCAACGGTCGGTCTTTTT
*sdiA*	GTCATCCCGTCCCCTTTAC	GGTTCGGCAACATCACAC
*luxR*	GATTGCTGCCCTCTGTTTTC	CGGCTTCTTCCAGTGAAT
*luxS*	CGACCACCTCAACGGTAA	GCACATCACGCTCCAGAATA
*QseB*	GCGAAAAGGGTAAACAGG	CGCAGTAAGAGTTCCAGCA
*csgA*	ATGCCCGTAAATCTGAAACG	ACCAACCTGACGCACCATTA
*csgB*	CGCATGTCGCTAACAAGGTA	ATTATCCGTGCCGACTTGAC
*csgD*	GATGGAAGCGGATAAGAAGC	GACTCGGTGCTGTTGTAGC
*fliZ*	CGGTTTCAAGCAGTATTTGT	CGGTAAAGGGGGATTTCTG
*flhD*	TTCCGCCTCGGTATCAAC	GCCGTATCGTCCACTTCATT
*motB*	AGTGGAAAAGCAGCCGAATA	GCAACCCCTCCTGAACTAAA

## Data Availability

Data is contained within the article.
